# Comparison of LC-MS and LC-DAD Methods of Detecting Abused Piperazine Designer Drugs

**DOI:** 10.3390/jcm11071758

**Published:** 2022-03-22

**Authors:** Anna Welz, Marcin Koba, Piotr Kośliński, Joanna Siódmiak

**Affiliations:** 1 Department of Toxicology and Bromatology, Faculty of Pharmacy, Collegium Medicum Nicolaus Copernicus University, 85-089 Bydgoszcz, Poland; kobamar@cm.umk.pl (M.K.); piotr.koslinski@cm.umk.pl (P.K.); 2 Department of Laboratory Diagnostics, Faculty of Pharmacy, Collegium Medicum Nicolaus Copernicus University, 85-094 Bydgoszcz, Poland; asiapollak@wp.pl

**Keywords:** piperazine designer drugs, benzylpiperazine derivatives, phenylpiperazine derivatives, LC-MS, LC-DAD, drugs of abuse, poisoning, misuse, psychoactive

## Abstract

Recreational use of piperazine designer drugs is a serious threat to human health. These compounds act on the body in a similar fashion to illegal drugs. They induce psychostimulatory effects as well as visual and auditory hallucinations to varying degrees. In many cases of poisoning and deaths, the presence of two or even several psychoactive substances have been demonstrated. Piperazine derivatives are often found in such mixtures and pose a great analytical problem during their identification. Additionally, some piperazine derivatives can be detected in biological material as a result of metabolic changes to related drugs. Therefore, it is necessary to correctly identify these compounds and ensure repeatability of determinations. This article presents a comparison of the methods used to detect abused piperazine designer drugs using liquid chromatography in combination with a diode-array detector (LC-DAD) or mass spectrometer (LC-MS). Each of methods can be used independently for determinations, obtaining reliable results in a short time of analysis. These methods can also complement each other, providing qualitative and quantitative confirmation of results. The proposed methods provide analytical confirmation of poisoning and may be helpful in toxicological diagnostics.

## 1. Introduction

The available literature and data indicate an increasing number and chemical diversity of new psychoactive substances (NPS), also known as designer drugs [1,2,3]. Products of this type are advertised as a modern alternative to illegal drugs, the possession and sale of which is prohibited by law [4,5]. The growing popularity of NPS, the possibility of online purchase and the large number of people experimenting with these compounds is a visible problem in Europe and around the world [6,7,8].

Piperazine derivatives from this group of designer drugs aroused interest due to their behavioral, neuroendocrine, psychostimulatory and hallucinogenic effects [9,10,11,12,13,14,15]. They appeared on the illicit drug market as modified analogues of narcotic drugs such as amphetamine [9,16,17]. The structural modifications of piperazine derivatives are also similar to the known amphetamine-derived compounds [18]. Chemically, these compounds are derived from piperazine, an organic heterocyclic compound with two nitrogen atoms in the opposite position [10,19,20]. Due to their structure, all piperazine derivatives can be divided into two groups: benzylpiperazines, e.g., N-benzylpiperazine (BZP), 1-(3,4-methylenedioxybenzyl)piperazine (MDBP), 1-(4-fluorobenzyl)piperazine (pFBP), 1,4-dibenzylpiperazine (DBZP); and phenylpiperazines, e.g., 1-(3-trifluoromethylphenyl)piperazine (TFMPP), 1-(3-chlorophenyl)piperazine (mCPP), 1-(4-parafluorophenyl)piperazine (pFPP), 1-(4-methoxyphenyl)piperazine (MeOPP). Table 1 shows the chemical structures of piperazine and the most common piperazine compounds in designer drugs.

Piperazine designer drugs show affinity for many 5-HT receptor subtypes [9,21,22]. Their hallucinogenic properties are the result of interaction with the 5-HT2A receptor, which may additionally lead to changes in the functioning of sensory processes [4,23]. High doses of BZP when bound to the 5-HT2 receptor, produce an effect approximately 10 times weaker than that of MDMA [24,25,26]. In addition, it increases the level of DA and NA, which leads to effects similar to those of amphetamine [27]. The simultaneous use of BZP with TFMPP or mCPP mimics the ecstasy profile, i.e., the levels of dopamine and serotonin increase [28]. The stimulant effects are a result of the action of BZP, and hallucinations have been observed after TFMPP [29]. Phenylpiperazine derivatives are sold together with BZP for enhanced effects on the body. Like other 1-arylpiperazines, MeOPP may exert central serotonergic effects [30]. It produces amphetamine-like effects, although it is less addictive [31]. MDBP shows a weak inhibition of serotonin reuptake and may cause slightly different effects compared to BZP [18]. Large doses of MDBP are needed to achieve the perceptible effects in recreational use.

The most frequently used doses of piperazine derivatives in recreational applications are: 50–1000 mg for BZP, 5–100 mg for TFMPP and 17.5–52.5 mg for mCPP [11,24,32,33,34,35,36,37]. For BZP and TFMPP used in combination, the reported ration was generally 2:1 [14,38]. The doses used for recreational purposes were about 50–100 mg for MDBP [18], and for MeOPP the dose was calculated as 1 mg/kg [30,39]. Piperazine designer drugs are most often sold in the form of colored tablets, capsules, powders, liquid mixtures and smoking forms [10,11,40,41]. Popular names of these products are: “Party pills”, “Retro pills”, “A2”, “Legal X”, “Legal E”, “X4”, “Herbal Ecstasy”, “Bliss”, “Combo”, “Lab-X”, “Cherries” and “Clear Light” [10,14,40,41]. Mixtures of piperazines with other psychoactive substances, such as MDMA, ketamine, amphetamines, cocaine, cannabis, diazepam (benzodiazepines) and ephedrine are also common [24,26,33]. They may also be contained in products advertised to potential users as ecstasy or amphetamines [14,19,29,41].

In their pharmacological profile, piperazine derivatives increase the level of dopamine (DA), serotonin (5-HT) and norepinephrine (NA), and block the neuronal uptake of these compounds [11,33]. Elevated levels of these neurotransmitters can cause a variety of desired as well as undesirable behavioral and clinical effects [2,21]. Recently, several evaluations of the cytotoxic effect of piperazine designer drugs were performed. These compounds have been shown to be potentially cardiotoxic [42,43], hepatotoxic [14,44], neurotoxic [38,45,46], nephrotoxic [33] and endocrine disrupting [10,47]. All piperazine designer drugs can result in dangerous health problems [10,19,26,40]. Even accidental ingestion can lead to severe poisoning or death [26,29,37].

In order to prove the involvement of piperazine designer drugs in the event of poisoning, information provided by clinicians about the symptoms of poisoning is required [19]. In clinical settings, toxicity is usually associated with the consequences of overdosing on recreational drugs [33]. Some symptoms may be specific to this group of compounds. Typically, there is tachycardia, hypertension and very characteristic pupil dilation [11,24,26,29,48]. Increased serotonin levels can cause life-threatening serotonin syndrome with changes in mental status (confusion, agitation, lethargy, coma), autonomic instability (hyperthermia, diaphoresis, vomiting, diarrhea) and neuromuscular disorders (tremor, muscle rigidity, myoclonus) [21,33,49]. Bruxism, trismus, headaches and dizziness, dissociative symptoms, as well as hyponatraemia, hyperkalaemia and metabolic acidosis are also observed [10,11,17,24,26,27,29,33,41]. Analytical evidence of the participation of piperazine designer drugs is needed for a comprehensive diagnosis [22]. This is all the more difficult as the concentrations in biological fluids may not be closely related to the clinical symptoms found [11,17,27,33]. According to the latest European Drug Report, compounds with piperazine are listed as substances that are harmful to human health and life [50]. There has also been an increase in the number of MDMA laboratories shut down by law enforcement agencies. Reduced ability to purchase MDMA products may result in increased use of piperazine designer drugs and more poisoning.

Piperazine derivatives belong to the basic chemical structures for the preparation of new compounds acting on the serotoninergic system [9]. Many studies have described the structure-activity relationship of large numbers of compounds with a chemical structure to the arylpiperazine side chain [9,51]. The attempts are still being made to develop metabolically stable derivatives as drug candidates [52].

The piperazine-based hallucinogenic and stimulant compounds are abused, yet not used therapeutically [9,27,33]. When identifying individual piperazine derivatives in biological material, it is necessary to analyze the circumstances of the event in order to correctly evaluate and interpret the result. Some piperazine designer drugs may be found in biological fluids as metabolites of related therapeutic drugs. The phenylpiperazine derivative, TFMPP is a metabolism product of antrafenine, an analgesic and anti-inflammatory drug [17,53]. TFMPP, as an antrafenine metabolite, easily enters the brain, reaching concentrations higher than in body fluids, and has a stimulating effect on 5-HT receptors [21,53]. Most probably the TFMPP may contribute to the therapeutic drug’s pharmacological effects [53]. In addition, antrafenine used to reduce inflammatory and neuroinflammatory pain as a cyclooxygenase inhibitor, could be a drug with possible use in the treatment of the advanced respiratory disease COVID-19 [54]. Identifying new targets for already approved drugs is one solution for treating viral diseases [54,55].

Another phenylpiperazine derivative, mCPP is the dominant metabolite of drugs such as: nefazodone, trazodone, etoperidone, mepiprazole, enziprazole [10,26]. Mepiprazole is a sedative drug and neuroleptic effects occur at doses in the higher range [56]. Trazodone is commonly used in the treatment of depression as a 5-HT2 receptors antagonist and a serotonin reuptake inhibitor [57,58,59,60]. The mCPP is the one of the main metabolism products of trazodone and is formed by nitrogen N-dealkylation [57,61]. It has been shown that mCPP as a reuptake transporter of serotonin and 5-HT2C agonist may possibly contribute to the anxiolityc and antidepressant trazodone effects [57]. With established patterns for trazodone use, sufficient mCPP concentrations can be achieved to identify this metabolite [62,63]. There are known reports of fatal cases following the deliberate overdose of trazodone [58,59,60]. The distinction between consumption of mCPP alone and consumption of related therapeutic drugs has a diagnostic character [62,64].

The phenylpiperazine derivative, MeOPP, is formed as a result of metabolic changes in the hypotensive drug urapidil [26]. Urapidil is an alpha-1-adrenoreceptor antagonist and additionally produces a sympatholytic effect and stimulates 5HT1A serotonin receptors, which are in the central nervous system [65]. MDBP is a derivative of benzilpiperazine and an active metabolite of the nootropic drug fipexide, which has been withdrawn from therapies due to severe side effects, like toxicity of the liver [66,67]. It has been observed that metabolites can sometimes be more toxic than the original drug [66]. It is believed that the covalent binding of active metabolites to proteins plays a major role in drug toxicity [66,68]. The distinction between recreational use of piperazine derivatives and consumption of related drugs is diagnostic, particularly in poisoning and fatal cases. Nowadays, prompt diagnosis is also necessary due to various comorbidities, especially during a pandemic [55].

There have been various reports of NPS-related poisoning and deaths where piperazines have been found [10,26,27,29,33,37,69]. However, the main issue is the lack of ability of laboratory confirmation of the occurrence of poisoning with piperazine designer drug involvement [10,26]. The underestimation of the number of poisoning cases may also be the result of the missing comprehensive analytical procedure in the detection of the designer drug piperazine in biological samples [70]. Additionally, the available immunoassays for known abused drugs cannot easily detect piperazines [11,16,17,62]. Wherever circumstances indicate drug use, the positive and negative test results should be confirmed by other techniques, as observed in studies with amphetamines [71,72]. Till now, various modern NPS detection methods, which use liquid chromatography (LC) or gas chromatography (GC), have been proposed [3,12,15,41,69,70,73,74,75,76,77,78]. However, other studies using GC-MS, LC-MS and LC-DAD usually did not deal directly with the piperazine designer drugs [12,15,69,75,76]. Widely used GC-MS technique is quite often chosen for systematic toxicological analysis (STA), although the preparation of samples of piperazine derivatives requires derivatization, which significantly extends the time of determinations [41,64,77]. LC-MS is seen as a complementary technique to GC-MS and can be successfully used to for the detection of unstable, low-dosed or polar drugs, specifically in biological fluids [79]. HPLC techniques with DAD detection have also been used in the past for screening purposes, allowing the detection of non-volatile and more polar compounds in gas chromatography and can be used for the successful detection of piperazine derivatives [41,79]. In addition, the relatively low cost of equipment and its operation allows for the availability of determinations in many laboratories. LC-MS becomes an increasingly commonly used apparatus, however, although many methods were published until now, piperazine designer drugs are not part of the routine approach in laboratory analysis [26]. It should be emphasized that piperazine designer drugs are compounds that cause serious toxicity even at regular, standard doses [19,26,33]. Often these compounds are used in mixtures with other known stimulants, which makes it very difficult to identify [24,29,33,37,70]. Analytical confirmation of piperazine designer drugs as main compounds causing the poisoning effect is required for medical interventions saving human health and life [29,33,37]. Therefore, there is an increasing demand for the use of instrumental techniques that enable the reliable and reproducible detection of designer piperazine drugs.

The article presents a comparison of the recently developed LC-MS method with the new LC-DAD method in terms of their ability to detect piperazine designer drugs. Using the LC-MS method, tested piperazine designer drugs can be identified on the basis of their precursor ion, specific product ions and measured retention time. The following deuterated analogs were used as preferred internal standards: BZP-D7; mCPP-D8; TFMPP-D4; which allowed us to obtain a high level of confidence in the results. Using the LC-DAD method, tested piperazine derivatives compounds can be recognized by their characteristic UV-VIS spectra, retention time and compliance with the standard. In the process of validating this method, the following parameters were assessed: measuring range, linearity, limit of detection (LOD), limit of quantification (LOQ) and method repeatability. A good separation was obtained for all tested analytes with a run time of 20 min, which confirms the appropriate selectivity of the method. Pentedrone was used as an internal standard, which was selected in an experimental way. The LC-DAD and LC-MS methods presented in the article allow for the independent detection of piperazine derivatives in non-biological and biological matrices, obtaining a good separation of the analytes in a short analysis time. The proposed analytical methods provide confirmation of poisoning with piperazine designer drugs and may be helpful in comprehensive toxicological diagnostics.

## 2. Materials and Methods

The method of detection abused piperazine designer drugs in biological material using LC-MS was the subject of a separate publication [22]. The present article lists the results and stages of the described methodology, which are the most important from the point of view of comparing the LC-MS and LC-DAD methods.

### 2.1. Reagents and Solvents

Standards used in the tests: 1-benzylpiperazine dihydrochloride (BZP), 1-(3,4-methylenedioxybenzyl)piperazine (MDBP), 1-(4-fluorobenzyl)piperazine (pFBP), 1-(3-chlorophenyl)piperazine hydrochloride (mCPP), 1-(3-trifluoromethylphenyl) piperazine hydrochloride (TFMPP), pentedrone hydrochloride and deuterated internal standards including BZP-D7, mCPP-D8 and TFMPP-D4 were ordered from Sigma-Aldrich (Darmstadt, Germany). BZP, mCPP, TFMPP and pentedrone hydrochloride were received as a 1 mg/mL standard in methanol. MDBP and pFBP were obtained from 10 mg of powder dissolved in methanol. BZP-D7, mCPP-D8 and TFMPP-D4 was purchased as deuterated internal standards at 100 µg/mL in methanol. Sodium phosphate monobasic dihydrate, 85% orthophosphoric acid, methanol hypergrade for LC-MS, acetonitrile hypergrade for LC-MS and formic acid for LC-MS were obtained from Sigma-Aldrich (Merck, Darmstadt, Germany). The sodium hydroxide was purchased from Avantor Performance Materials Poland S.A. (formerly POCH). Filtered water was obtained from the demineralizer HLP 5UV Hydrolab (Straszyn, Poland). All biological samples planned for fortification (serum, urine) were collected from healthy volunteers after receiving their informed consent. This study has obtained the consent of the Bioethical Committee of the Nicolaus Copernicus University in Toruń and the Collegium Medicum in Bydgoszcz (consent number: KB 467/2018).

### 2.2. Instrumentation

Analyses of piperazine derivatives were performed using the Shimadzu LCMS-8045 triple quadrupole liquid chromatograph mass spectrometer (LC-MS) equipped with a heated ESI probe or using high-performance liquid chromatography (HPLC) system Shimadzu-Nexera XR combined with SPD-M20A prominence photodiode array detector (DAD). Both systems were operated with LabSolution software. Common components of Shimadzu-Nexera XR HPLC were as follow: LC-20ADXR liquid chromatograph pump, SIL-20ACXR autosampler and CTO-20AC prominence column oven. The degassing units DGU-20A3R and DGU-20A5R were used, respectively.

### 2.3. Chromatographic Conditions for LC-MS

Chromatographic separation of the tested substances was performed on a Synergi 4 μm, Hydro—RP, 80A, C18 with polar endcapping, 150 × 2.00 mm LC column (Phenomenex, Inc., Torrance, CA, USA), in reversed-phase mode, with a mobile phase gradient. The mobile phase consisted of a mixture of water with addition of 0.1% formic acid (mobile phase A) and methanol with the addition of 0.1% formic acid (mobile phase B). The mobile phase flow was 0.5 mL/min and took place in a gradient system: 0–2 min—10% B, 8 min—100%, 9 min—10% B and 15 min—10% B. The injections were performed by an autosampler and the volume of injection was 5 μL. The column was thermostated at 30 °C and the total run time was 15 min. In the conducted analyses electrospray ionization in the positive mode has been used. The values of the working parameters of the mass spectrometer were as follows: the heater block temperature was 400 °C, the desolvation line (DL) temperature was 250 °C, the nebulizing gas flow was 3 L/min, the drying gas flow was 10 L/min and heating gas flow was 10 L/min. Dynamic multiple reaction monitoring (MRM) mode was used and two MRM transitions were selected for each compound [22].

### 2.4. Chromatographic Conditions for LC-DAD

Chromatographic separation was carried out on a Xterra RP C18 5 μm; 4.6 × 150 mm column (W21611A Waters Corporation, Milford, MA, USA), in reversed-phase mode, with a mobile phase gradient. The components of the mobile phase were: 20 mM phosphate buffer solution (mobile phase A), LC-grade acetonitrile (mobile phase B) and LC-grade methanol (mobile phase C). The flow rate of the mobile phase was set to 0.6 mL/min, in which the starting condition was 85% phosphate buffer/10% methanol/5% acetonitrile. After 3 min isocratic flow, the elution proceeded with the following gradient: 8–16 min—70%BF/20%MetOH/10%ACN; 17 min—85%BF/10%MetOH/5%ACN, followed by 3 min equilibration. The total analysis time was 20 min. The column oven temperature was 40 °C. An autosampler was used to inject the sample and the volume of injection was 10 μL. Chromatograms of piperazine derivatives were recorded in the spectral range from 200 to 300 nm. Fresh phosphate buffer was prepared each time for the tests. The concentration of the phosphate buffer and the proportions of the mobile phase were developed experimentally. Analyses were performed at pH 2.7, 3.6, 4.1, 4.6 and 6.0, respectively. Phosphate buffer (BF) with a concentration of 20 mM and pH 4.1 corrected to this value with 85% phosphoric acid (H_3_PO_4_) and 1M NaOH was used for the tests.

### 2.5. LC-MS Analysis—Preparation of Calibration Samples

Dilutions of the piperazine derivatives BZP, MDBP, pFBP, mCPP and TFMPP were carried out with the methanol. A stock solution of internal standards was prepared by combining by deuterated compounds: BZP-D7, mCPP-D8, and TFMPP-D4.

### 2.6. Biological Samples Preparation

Urine and serum samples were divided in portions of 100 μL. Internal standards and piperazine derivatives, both at appropriate concentrations were added. The samples were then alkalized with 3M NaOH, then cold acetonitrile was added, vortexed and finally centrifuged at 10.0 rpm for 5 min. The received supernatants were additionally filtered through a PES (polyethersulfone) membrane filter (ø = 25 mm, with pore size 0.45 μm) into a vial. The analysis of piperazine derivatives were performed using liquid chromatography coupled with a diode detector (LC-DAD) or a mass spectrometer (LC-MS).

### 2.7. LC-DAD Analysis—Preparation of Samples for Calibration

All dilutions of BZP, MDBP, pFBP, mCPP, TFMPP and Pentedrone stock solutions were prepared by serial dilutions with methanol. The linearity of the method was checked using concentrations of tested compounds as follows: 0.5, 1, 2, 3, 4, 5, 6 and 7 μg/mL. Pentedrone was used as an internal standard and it was added to each sample in a concentration of 0.5 μg/mL. The prepared samples were analyzed in quadruplicate.

### 2.8. LC-DAD Analysis—Validation of the Method

The validation process of the described method evaluates parameters like: measuring range, linearity, repeatability of the method, limit of detection (LOD), limit of quantification (LOQ) and the use of an internal standard.

### 2.9. LC-DAD Analysis—Linearity of the Method

The linearity range of the developed method was determined on the basis of a calibration curve calculated for each compound (in the measured range from 0.5 to 7 μg/mL). Adopted calibration levels were: 0.5 μg/mL, 1 μg/mL, 2 μg/mL, 3 μg/mL, 4 μg/mL, 5 μg/mL, 6 μg/mL and 7 μg/mL. An internal standard was added at a concentration of 0.5 μg/mL. The dependence of the analyte concentration as a function of the ratio of the analyte peak area to the internal standard peak area was plotted on the basis of the results obtained. The Microsoft Excel software was used for data analysis. The regression equations were obtained for individual compounds and the coefficient of linear determination R2 were determined.

### 2.10. LC-DAD Analysis—Analytical Limits

The tests were performed for ten replicate concentrations of analytes close to the predicted limit of detection. The limit of detection (LOD) was determined from the obtained data. The limit of quantification (LOQ) value was calculated from the following equation LOQ = 3 LOD.

### 2.11. LC-DAD Analysis—Method Repeatability

The reproducibility of the method was assessed by using control samples at three empirically determined concentration levels of piperazine designer drugs in the linear range of the calibration curve. The reproducibility of peak areas of the piperazine derivatives and the retention times were assessed within the day and between different days.

## 3. Results

### 3.1. LC-MS Method—MRM Transitions and Chromatographic Separation

**Figure 1:**
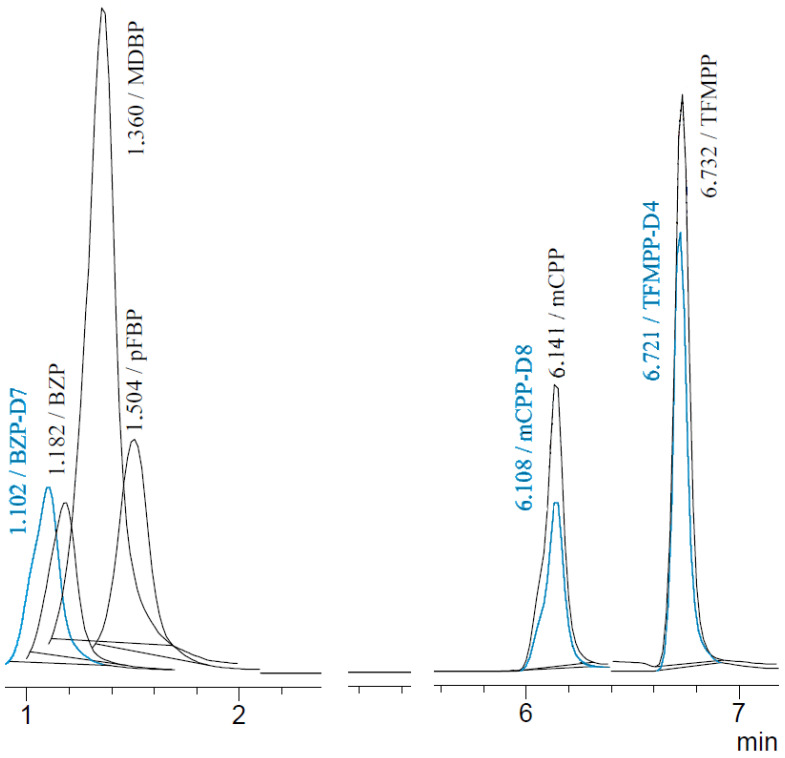
Chromatogram of piperazine derivatives (and deuterated analogues) obtained by LC-MS method with corresponding retention times: BZP (1.182), BZP-D7 (1.102), MDBP (1.360), pFBP (1.504), mCPP (6.141), mCPP-D8 (6.108), TFMPP (6.732) and TFMPP-D4 (6.721).

A good chromatographic separation of tested compounds was obtained under the above-mentioned chromatographic conditions. An exemplar chromatogram of the tested piperazine derivatives is shown in Figure 1 (intensity versus retention time).

Detailed method description and results obtained, such as measures of standards and biological samples, and validation parameters, e.g., regression equations, linear ranges, determination coefficient, analytical limits, quality control parameters, are described in the separate report [22]. The LC-MS method also confirmed the reproducibility of fragmentation for the tested compounds. Table 2 shows chemical structures, [M + H]^+^ and major fragmentation patterns of piperazine designer drugs detected by mass spectrometry.

### 3.2. LC-DAD Method—UV-VIS Spectra and Chromatographic Separation

**Figure 2:**
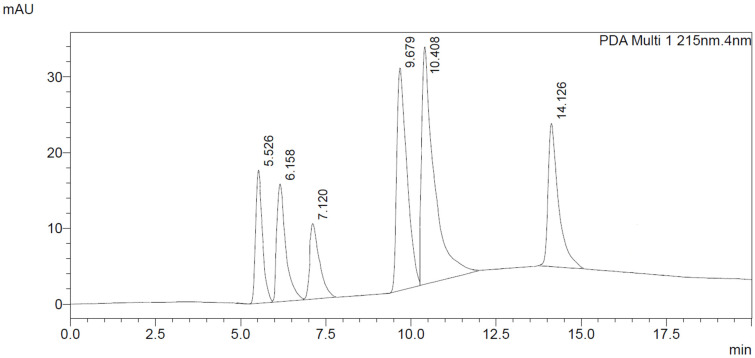
Chromatogram of piperazine derivatives (and pentedrone as internal standard) obtained by HPLC-DAD method with corresponding retention times: BZP (5.5), MDBP (6.1), pFBP (7.1), mCPP (10.4), TFMPP (14.1) and pentedrone (9.6).

In the determination of piperazine derivatives by the LC-DAD method, all work steps have been optimized. A good chromatographic separation of piperazine derivatives was obtained by using an Xterra RP C18 column. The LC-DAD chromatogram of tested piperazine derivatives obtained with this method is shown in Figure 2 (intensity versus retention time).

**Figure 3:**
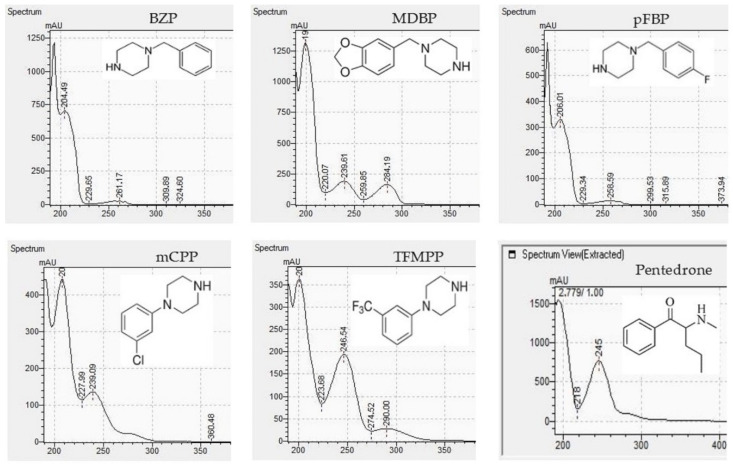
UV-VIS spectra of benzyl- and phenyl derivatives of piperazine and pentedrone.

The appropriate chromatographic column was selected and the chromatographic conditions were optimized. The chromatography has been optimized with an eluent gradient, obtaining a good peak shape and good separation of analytes. The analysis time is 20 min, which includes an equilibration of column. UV-VIS spectra, retention times and compliance with the standard were obtained for all tested compounds. Characteristic UV-VIS spectra of piperazine and pentedrone derivatives are shown in Figure 3. Pentedrone as an internal standard was chosen in an experimental way.

Table 3 and Table 4 summarize exemplary retention times of compounds depending on the pH of the phosphate buffer (20 mM) for two different mobile phase compositions. Table 3 presents data for the mobile phase with the composition: BF70/MeOH20/ACN10, and Table 4 presents data for the mobile phase with the composition: BF85/MeOH10/ACN5.

### 3.3. Validation of the LC-DAD Method (Measuring Range, Linearity, Repeatability, LOD and LOQ)

The applied analysis conditions proved to be suitable for the separation of the tested piperazines. An eight-point calibration curve was developed (*n* = 8). Linearity was obtained in the proposed calibration range at high values of the coefficient of determination, varying between 0.9917 and 0.9984. The collected results were used to determine the regression equation. The analytical limits and all results are summarized in Table 5.

The reproducibility of the method was checked by performing determinations of control samples (quality control, QC) at three concentration levels (LQC, MQC, HQC). The results were used to calculate the coefficients of variation in retention times and peak areas. A summary of these results is presented in Table 6. Test results conform to SWGTOX guidelines [80].

The developed methods were used to determine piperazine compounds in enriched biological samples. Qualitative identification was performed. The results of the qualitative tests are presented in Table 7. The characteristic UV-VIS spectra are consistent with the standards of piperazine derivatives and confirm their presence in biological material.

The quantitative analysis of piperazine derivatives was performed by LC-MS method and the results are presented in Table 8.

## 4. Discussion

The recently developed LC-MS method and the new LC-DAD method were compared for their ability to detect the designer drugs piperazine. For each method, optimal analysis conditions were developed.

### 4.1. LC-MS Method

The analysis time using the LC-MS method was 15 min. All test compounds were identified by designating a precursor ion and two product ions at the appropriate retention time. As internal standards, deuterated analogues such as BZP-D7, mCPP-D8 and TFMPP-D4 were used. Discussion about the results obtained, and a detailed method description are presented in the separate report [22].

### 4.2. LC-DAD Method

In the presented studies, analyses were performed using the buffer concentrations of 10 mM, 20 mM and 100 mM. Using a concentration of 10 mM, the obtained results were not satisfactory. On the other hand, using 100 mM, individual compounds were determined, however, it was not possible to separate the mixture of benzyl and phenylpiperazine from one sample. The best results of the chromatographic separation were obtained for the concentration of 20 mM for both individual compounds and the mixture.

A very important task was to select a proper pH of the buffer. The analyses were performed at pH 2.7; 3.6; 4.1; 4.6 and 6.0, respectively. Results are presented in Table 3 and Table 4 in previous section.

It can be observed from the presented results that in the case of benzylpiperazine derivatives, the change in pH has a significant impact on the retention time of these compounds. The higher the pH, the longer the retention time of the benzylpiperazine derivatives is. In the case of phenylpiperazine derivatives, the change in pH has little effect on the retention time of these compounds. For the extension of the retention time of the phenylpiperazine derivatives, the percentage of the components of the mobile phase was of the greatest importance.

Pentedrone as an internal standard was chosen in an experimental way. It is a chemical compound from the group of ketoarylamines. This compound has chemical and physical properties similar to piperazine derivatives, it is well separated from the tested analytes, and the retention time is similar to the retention times of the components present in the sample [81]. A series of tests were carried out in order to find an internal standard, which, while differing from piperazine derivatives, will also be measurable under the developed conditions. From the group of ketoarylamine derivatives, the following were also analyzed: Butylon; Bufedrone; Flephedrone; MDPV; and Metedron. In addition, selected synthetic cannabinoids were also analyzed: AM 694; JWH 250; UR 144; and XLR 11. Nevertheless, for the compounds listed above, no satisfactory results were obtained. It is also a confirmation that the developed method is mainly aimed at the detection of piperazine derivatives. A separate detection method has been developed for synthetic cannabinoids and ketoarylamine derivatives, however this is beyond the scope of this article.

### 4.3. Comparison of LC-MS and LC-DAD Methods

For each of these methods, optimal analysis conditions were developed. Elements of the measurement systems and operating parameters are presented in Table 9.

Table 10 presents the characteristic MS and UV-VIS spectra of the tested piperazine designer drugs.

The presented methods made it possible to identify piperazine designer drugs on the basis of the consistency of the retention times of the analytes present in the test sample and in the reference sample. The UV-VIS spectra of piperazine derivatives differ from each other, and they have absorption maxima at different wavelengths. These features were used to confirm the presence of the tested compounds in biological material. In turn, for the LC-MS method for the tested compounds, two MRM transitions were monitored for specific quantification. Such an analytical process allows for the elimination of disturbances from the biological matrix [16]. The designer piperazine drugs have structure-related fragmentation properties, which can be assessed in the analyses [22,79]. The study of the characteristic fragments of molecules, combined with the determination of their exact masses, can be of great help in identifying unknown samples [78]. For quantitative determinations of piperazine derivatives in the LC-MS method, analogues labelled with a stable isotope (SIL) were used [22,82,83,84,85]. This allowed the correction of errors caused by matrix effects [16,81,85]. Alkalization of biological samples allowed for the capture of reliable results [22]. The simple processing of biological samples enabled very good results and significantly shortened the working time. In poisonings caused by piperazine, concentrations have been recorded that can be detected by LC-DAD and quantified by LC-MS. In earlier studies, piperazine designer drugs were detected in serum in the concentration ranges of 15–585 ng/mL and in urine from 0.40 μg/mL to 202 μg/mL [10,26,29,37]. The obtained values of LOD and LOQ of the LC-MS method indicate the possibility of detecting piperazine designer drugs at the level of single ng and event amount at the level of pg [22]. Using the LC-DAD method, piperazines can be detected in the ng and μg range. This technique can be successfully used for screening as poisoning is usually associated with high levels of toxic substances. Unlike other advanced technologies, LC-DAD is also easier to use, which makes the work much easier.

The benefit of the LC-MS method used is the high sensitivity of the determinations. On the other hand, the advantage of the LC-DAD method is the high repeatability of the results. Processing the sample without the need for derivatization significantly simplifies and shortens the analysis time, especially compared to the methods, which are based on GC-MS [41,77]. The short time of the analysis of the serum or urine samples will allow us to assess the current health status of the patient, as opposed to the analyses carried out, i.e., in the hair matrix [69,70]. Until now, other studies using LC-MS, GC-MS, and LC-DAD did not explicitly target the compounds from the tested group [12,15,69,75,76]. The methods presented in the article may complement each other for the research on piperazines or they may be used independently.

Difficulty in assessing the results obtained can be due to the fact that the metabolic processes of related therapeutic drugs may result in the detection of 1-aryl-piperazines in the biological matrices [86]. Piperazine derivatives, detected as metabolic products, account for about 10% of the applied dose. The great advantage of the proposed methods is that they can be used to monitor 1-aryl-piperazines as metabolites of therapeutic drugs. Monitoring concentrations of piperazine derivatives as metabolites could contribute to the safety of the treatment. The Table 11 shows the piperazine derivatives as metabolites of therapeutic drugs.

The identification of piperazine designer drugs is also necessary to predict interactions and inter-individual differences in pharmacokinetic profiles. Some homologous cytochrome P450, CYP2D6 and COMT (catechol-O-methyltransferase) enzymes catalyze many drugs, including the metabolism of piperazines [33]. These isoenzymes can differ in amino acid sequence, which can cause side effects, especially when MDMA is used concomitantly [9,33]. The metabolism of the piperazine designer drugs may indicate a problem of interaction with other drugs undergoing similar transformation [26]. Inhibitors of this metabolic pathway can simultaneously potentiate the effects of piperazines leading to dangerous health effects [79]. For example, the inhibitor of CYP2D6, thioridazine may increase the plasma concentration of mCPP [9,26]. The diagnostic process in clinical toxicology is based on the recognition or the definitive ruling out of acute or chronic poisoning [79]. Confirmation of the identity of compounds causing the poisoning is essential in saving lives.

## 5. Conclusions

Piperazine designer drugs are abused synthetic stimulants. These compounds are seen by users as alternatives to MDMA and amphetamines due to their similar effects on the central nervous system. The recreational use of piperazine derivatives can result in acute or chronic poisoning. The article describes methods using liquid chromatography techniques for the independent detection of piperazine designer drugs in biological and non-biological matrices. The benefit of the LC-MS method is the high sensitivity of determinations, while the LC-DAD method ensures high reproducibility of results. The LC-MS method also confirmed the reproducibility of the main fragmentation patterns for the tested compounds. The addition of deuterated analogues as internal standards to the tested samples ensured reproductible quantification. The characteristic UV-VIS and MS spectra were used to confirm the presence of the tested compounds in the biological material. The suitability of these methods for the evaluation of 1-aryl piperazines as metabolites of parent therapeutic drugs can be investigated in the future.

The presented methods enable the detection of piperazine designer drugs in a different concentration range and additionally in a short time of analysis. Rapid analytical confirmation of the cause of poisoning is essential in medical interventions that save human health and life. The proposed methods may be useful techniques in situations requiring analytical confirmation of piperazine designer drug poisoning and may be helpful in comprehensive toxicological diagnostics.

## Figures and Tables

**Table 1:** Chemical structures of piperazine and piperazine designer drugs.

**PIPERAZINE** 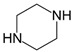	
**Benzylpiperazine Derivatives**	**Phenylpiperazine Derivatives**
BZP N-benzylpiperazine 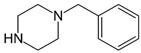	mCPP 1-(3-chlorophenyl)piperazine 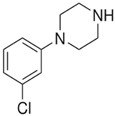
MDBP 1-(3,4-methylenedioxybenzyl)piperazine 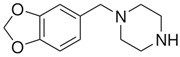	TFMPP 1-(3-trifluoromethylphenyl)piperazine 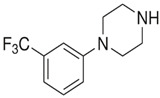
pFBP 1-(4-fluorobenzyl)piperazine 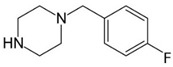	pFPP 1-(4-parafluorophenyl)piperazine 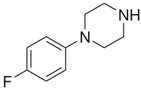
DBZP 1,4-dibenzylpiperazine 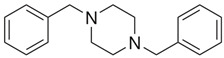	MeOPP 1-(4-methoxyphenyl)piperazine 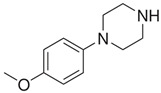

**Table 2:** Chemical structures, precursor ions, [M + H]^+^ and fragmentation patterns of piperazine designer drugs observed in LC-MS.

Compound	Precursor Ion (*m*/*z*)	Fragmentation Patterns (*m*/*z*)		
BZP 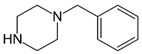	177.3 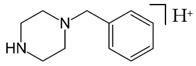	90.95 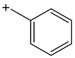	64.95 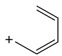	86 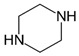
C_11_H_16_N_2_	[C_11_H_16_N_2_]^+^	[C_7_H_7_]^+^	[C_5_H_5_]^+^	[C_4_H_10_N_2_]
MDBP 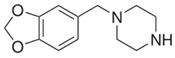	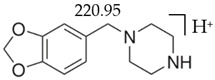	135.00 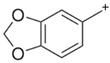	76.9 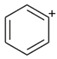	86 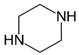
C_12_H_16_N_2_0_2_	[C_12_H_16_N_2_0_2_]^+^	[C_8_H_7_O_2_]^+^	[C_6_H_5_]^+^	[C_4_H_10_N_2_]
pFBP 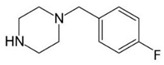	195 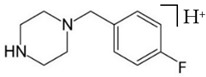	108.90 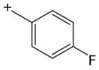	83.00 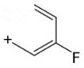	86 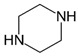
C_11_H_15_FN_2_	[C_11_H_15_FN_2_]^+^	[C_7_H_6_F]^+^	[C_5_H_4_F]^+^	[C_4_H_10_N_2_]
mCPP 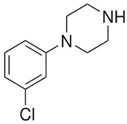	197.05 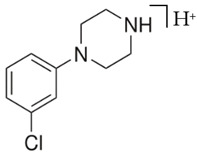	153.95 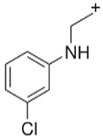	117.95 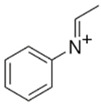	44 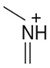
C_10_H_13_ClN_2_	[C_10_H_13_ClN_2_]^+^	[C_8_H_9_ClN]^+^	[C_8_H_8_N]^+^	[C_2_H_5_N]^+^
TFMPP 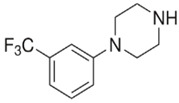	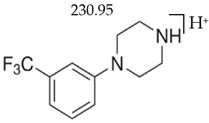	187.95 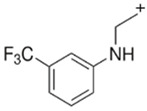	118.10 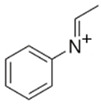	44 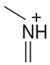
C_11_H_13_F_3_N_2_	[C_11_H_13_F_3_N_2_]^+^	[C_9_H_9_F_3_N]^+^	[C_8_H_8_N]^+^	[C_2_H_5_N]^+^

**Table 3:** Exemplary retention times (t_R_) for piperazine derivatives depending on the pH of the phosphate buffer using the mobile phase with the percentage composition: BF70/MeOH20/ACN10.

Compound Name	Mobile Phase Composition: BF70/MeOH20/ACN10					
	BF 20 mM					BF 100 mM
	t_R_ (pH 2.7)	t_R_ (pH 3.6)	t_R_ (pH 4.1)	t_R_ (pH 4.6)	t_R_ (pH 6.0)	t_R_ (pH 6.0)
BPZ	3.036	3.659	3.832	3.857	4.032	3.985
MDBP	3.065	3.775	3.979	4.009	4.187	4.135
pFBP	3.359	4.192	4.392	4.423	4.632	4.560
mCPP	5.215	5.235	5.309	5.320	5.736	5.591
TFMPP	8.216	8.249	8.368	8.561	9.707	9.382

**Table 4:** Exemplary retention times (t_R_) for piperazine derivatives depending on the pH of the phosphate buffer using the mobile phase with the percentage composition: BF85/MeOH10/ACN5.

Compound Name	Mobile Phase Composition: BF85/MeOH10/ACN5				
	BF 20 mM				BF 100 mM
	t_R_ (pH 3.6)	t_R_ (pH 4.1)	t_R_ (pH 4.6)	t_R_ (pH 6.0)	t_R_ (pH 6.0)
BPZ	4.996	5.702	6.024	6.472	6.588
MDBP	5.437	6.438	6.859	7.477	7.534
pFBP	6.417	7.380	7.775	8.340	8.433
mCPP	11.763	11.835	12.015	12.654	12.791
TFMPP	23.663	23.680	24.569	26.783	27.257

**Table 5:** Summary of results and validation parameters for LC-DAD method.

Analytes	Internal Standard	Linear Range (ng/mL)	Regression Equation	R^2^	Analytes LOD (ng/mL)	Analytes LOQ (ng/mL)
BZP	Pentedrone	500–7000	y = 0.0001x − 0.0235	0.9984	150	450
MDBP	Pentedrone	500–7000	y = 0.0006x − 0.0397	0.9917	110	330
pFBP	Pentedrone	500–7000	y = 0.0027x − 0.3238	0.9941	100	300
mCPP	Pentedrone	500–7000	y = 0.0005x + 0.0645	0.9919	150	450
TFMPP	Pentedrone	500–7000	y = 0.0006x − 0.0884	0.9961	140	420

**Table 6:** Repeatability for the LC-DAD method expressed as the coefficient of variation (CV) in the retention times (t_R_), the surface area of the tested piperazines during the day and between days.

Analytes	Level	Daily Accuracy for t_R_, *n* = 12 CV (%)	Daily Accuracy for AUC, *n* = 12 CV (%)	Accuracy between Days for t_R_ CV (%)	Accuracy between Days for AUC CV (%)
BPZ	LQC	0.24	1.42	0.64	9.64
	MQC	0.14	1.18	0.49	5.04
	HQC	0.12	1.56	0.44	7.75
MDBP	LQC	0.23	3.12	0.38	4.06
	MQC	0.14	2.20	0.47	3.51
	HQC	0.11	1.20	0.40	5.07
pFBP	LQC	0.11	1.74	0.26	10.31
	MQC	0.03	0.76	0.05	5.73
	HQC	0.07	1.92	0.11	1.69
mCPP	LQC	0.04	0.95	1.32	2.24
	MQC	0.04	1.07	1.27	1.20
	HQC	0.04	1.00	1.34	2.47
TFMPP	LQC	0.05	2.34	0.84	4.65
	MQC	0.34	1.13	1.26	3.11
	HQC	0.04	1.20	1.44	3.47

**Table 7:** Confirmation of the presence of piperazine designer drugs in biological material (urine, serum).

Identification of Piperazine Designer Drug in Urine	Identification of Piperazine Designer Drugs in Serum
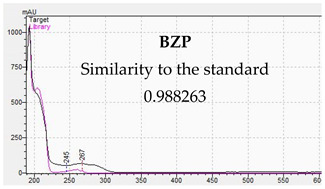	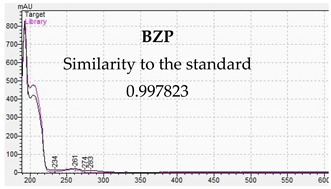
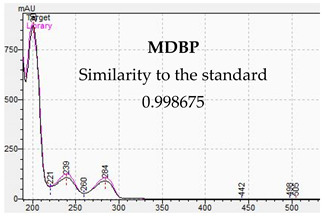	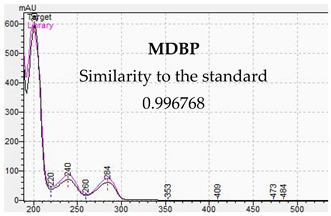
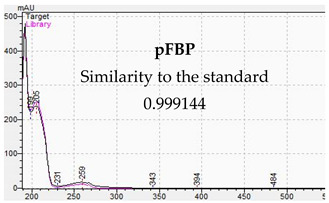	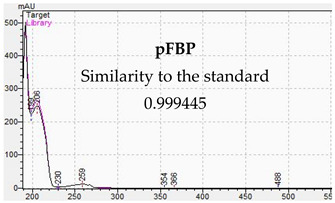
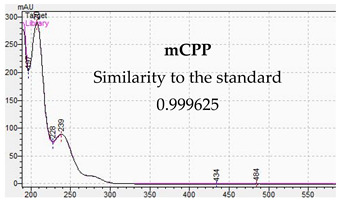	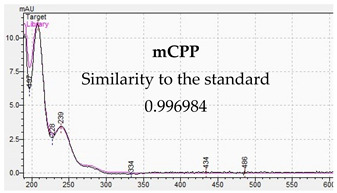
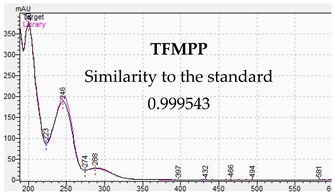	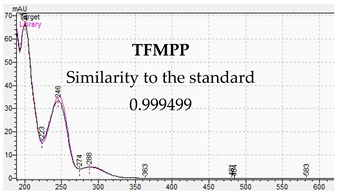

**Table 8:** Data obtained from the piperazine designer drugs analysis (urine, serum).

Analytes	Internal Standard		Urine			Serum	
		Average 1000 ng	Standard Deviation	%CV	Average 1000 ng	Standard Deviation	%CV
BPZ	BZP-D7	1183.70	14.47	1.18	1120.88	9.71	0.87
MDBP	BZP-D7	1021.15	7.70	0.75	983.20	11.25	1.14
pFBP	BZP-D7	973.34	13.08	1.34	1006.45	16.52	1.64
mCPP	mCPP-D8	1095.33	8.11	0.74	1146.73	18.71	1.63
TFMPP	TFMPP-D4	996.96	1.61	0.16	1013.00	27.61	2.73

**Table 9:** The conditions of the chromatographic analysis for the determination of piperazine designer drugs.

Elements of the Measuring System and Work Parameters	LC-MS Method	LC-DAD Method
Liquid chromatograph	LCMS-8045, Shimadzu	LC-DAD, Shimadzu
Mobile phase	A: Water (0.1%FA) B: Methanol (0.1%FA)	A: 20 mM phosphate buffer B: Acetonitrile C: Methanol
Column	Synergi Hydro-RP C18 4 μm; 2.00 × 150 mm	Xterra RP C18 5 μm; 4.6 × 150 mm
Injection volume	5 μL	10 μL
Analysis time	15 min	20 min

**Table 10:** Mass spectra and UV/VIS spectra of selected piperazine designer drugs.

Compound	Mass Spectra from LC-MS	UV/VIS Spectra from LC-DAD
BZP	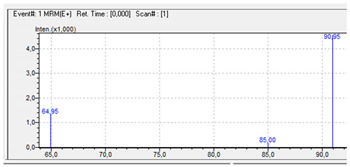	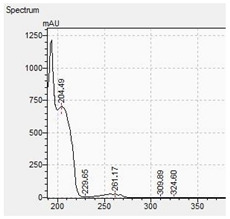
MDBP	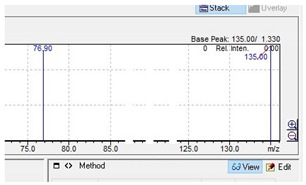	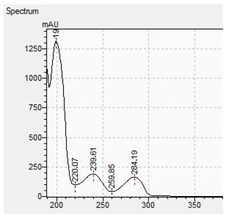
pFBP	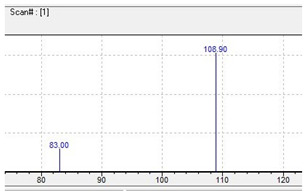	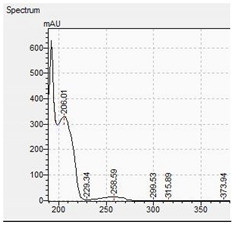
mCPP	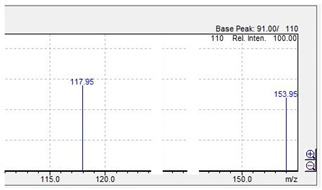	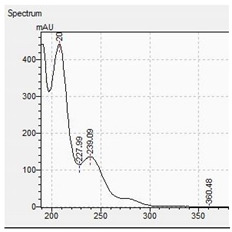
TFMPP	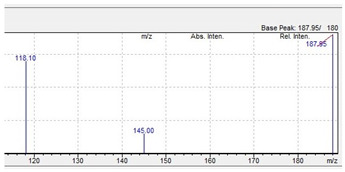	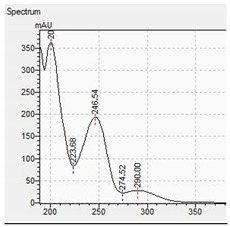

**Table 11:** Piperazine derivatives as metabolites of therapeutic drugs.

General Name of Therapeutic Drug	Pharmacological Classification	Piperazine Derivatives as Metabolite	References
Antrafenine	Analgesic	TFMPP	[17,53,54]
Trazodone	Antidepressant	mCPP	[57,58,59,60,62]
Nefazodone	Antidepressant	mCPP	[10,26]
Etoperidone	Antidepressant	mCPP	[10,26]
Enziprazole	Antidepressant	mCPP	[10,26]
Mepiprazole	Tranquilizer	mCPP	[10,26,56]
Urapidil	Antihypertensive	MeOPP	[26,65]
Fipexide (withdrawn from the treatment)	Nootropic	MDBP	[66,67]
